# Digital Rectal Examination: Perspectives on Current Attitudes, Enablers, and Barriers to Its Performance by Doctors-in-Training

**DOI:** 10.7759/cureus.40625

**Published:** 2023-06-19

**Authors:** Mary Teoh, Daniel Lee, David Cooke, Munyaradzi G Nyandoro

**Affiliations:** 1 General and Colorectal Surgery, Sir Charles Gairdner Hospital, Perth, AUS; 2 General Surgery, Fiona Stanley Hospital, Perth, AUS; 3 General and Colorectal Surgery, Fiona Stanley Hospital, Perth, AUS

**Keywords:** barriers, enablers, attitudes, doctors-in-training, digital rectal exam

## Abstract

Background

Digital rectal examination (DRE) is a valuable diagnostic tool for diagnosing multiple conditions, but its use has declined in practice. This study sought to provide perspectives on current attitudes, enablers, and barriers to performing DRE for doctors-in-training (DiTs) and explore strategies to improve and facilitate consistent, efficient, and effective execution of DRE.​​​​​​​

Methodology

Self-reported DRE practice among DiTs (n = 1,652) across three metropolitan health service regions in Western Australia was surveyed using a de-identified multiple-response ranking, dichotomous quantitative and qualitative survey. Data were analyzed using SPSS version 27 (IBM Corp., Armonk, NY, USA).

Results

A total of 452 (27%) DiTs responded to the survey, with an even distribution of key demographics between regions and specialties. The median post-graduate year was 2. Half of DiTs reported being comfortable with performing DRE. Most had training in medical school (71%), while 9.7% had no training in DRE. Chaperone availability, perceived invasiveness, and lack of confidence were key barriers; key enablers were formal training and senior colleague/departmental support.

The multivariate logistic regression showed that DiTs who reported being comfortable in performing DRE were significantly and independently associated with being a high-volume practitioner (p < 0.001), confident in diagnosing benign (p < 0.001) or malignant pathology (p < 0.001), perceived adequate DRE training (p < 0.001), prior formal DRE training (p = 0.007), and surgical subspeciality interest (p = 0.030).

Conclusions

Low levels of confidence and comfort in the performance of DRE among DiTs have resulted in the underutilization of a critical diagnostic tool. Future curriculum and departmental clinical practice interventions should address barriers while promoting enablers.

## Introduction

Digital rectal examination (DRE) is a valuable diagnostic tool forming part of a complete physical examination of the gastrointestinal and urogenital systems, providing vital information on various pathologies [1-4]. These can include general surgical (rectal cancer, hemorrhoids, fistulas), gastroenterological (detection of bleeding such as melaena or hematochezia, assessment of fecal incontinence or constipation), urological (prostate cancer assessment), and gynecological (pelvic floor prolapse or pelvic inflammatory disease) in nature. Furthermore, DRE is a valuable tool in assessing the anal tone in some spinal orthopedic or neurological disorders and is a key part of a multi-trauma secondary survey [1-4].

Failure to perform an adequate examination can lead to missed opportunities for early intervention, as evidenced by the case that led to this study’s conception [5]. The published case report highlighted a series of assumptions by independent treating clinicians of hemorrhoidal bleeding in the context of known hemorrhoids. This resulted in a diagnostic delay that impacted the timely management of anal squamous cell carcinoma (SCC). Historically, surgical teaching has been that the absolute contraindication to DRE is only when the examiner has no fingers or the patient has no anus [3]. Relative contraindications include the presence of an anal fissure and in children. Nevertheless, an inspection is essential and informative [3].

Modern clinical medicine has seen a decline in the use of DRE despite its established role as an aid to the diagnostician [3]. Studies have identified that the lack of training and time or the perception that DRE is of little clinical yield has rendered this physical examination obsolete [6-8]. These studies report that most of these examinations are often performed by junior doctors with minimal training and experience with DRE, an issue shared in the medical school curriculum. Medical students often have this vital examination poorly taught in a curriculum with minimal supervision and a lack of exposure during training [8].

In everyday practice, it is not unreasonable to expect that by internship, doctors-in-training (DiTs) should be able to recognize common benign conditions such as thrombosed hemorrhoids, fistula-in-ano, fissure, perianal abscess, melena, benign prostatomegaly, as well as malignant conditions such as low rectal cancer and prostate cancer. In comparison, less common conditions such as perianal SCC, melanoma, extramammary Paget’s disease of the anal canal, and prolapse might be challenging for a junior DiT to diagnose clinically. Given that education and experience with DRE are variable, this paper aimed to provide perspectives on the current attitudes, enablers, and barriers to performing DRE for DiTs.

This article was previously presented as a meeting abstract at the 2021 Royal Australasian College of Surgeons Annual Scientific Congress (ASC) on May 11th, 2021, in Melbourne and May 6th, 2019, in Bangkok.

## Materials and methods

Study population

In Australia, the natural progression of the medical career begins as an intern, then a resident, registrar, fellow of a specialty college, and, finally, a consultant. An intern is a post-graduate year one (PGY1) doctor with provisional registration. A resident is at least PGY2 and possesses general registration. Both interns and residents are supervised by registrars (usually PGY3 and above) who could either be accredited by a specialty college undergoing training or unaccredited.

All 1,652 DiT Interns (n = 322), Residents (n = 761), Registrars (n = 484), and Fellows (n = 85) from three metropolitan regions were invited via multimedia formats (email, hyperlinks, and QR codes on societies’ social media sites and at grand rounds and teaching sessions) to participate in an online, de-identified online survey. The self-reported DRE practice Survey Monkey™ was conducted between September 2018 and March 2019 and contained a mix of dichotomous (yes/no), multiple-response, and free-text completion items (Appendices). Multi-platform reminders were sent to improve the response rate. Essential components such as participants’ comfort and confidence levels with performing DRE were assessed using a five-point Likert scale. In addition, an internal validity question (likelihood of performing a DRE on a patient presenting with chest pain) was added to the survey questions.

The low-volume practice was defined as performing less than 20 DREs per year, while the high-volume practice was 21 or more. Functional conditions were defined per Talley and O’Connor’s clinical examination of the pelvic floor using special tests for pelvic floor dysfunction in a four-step assessment (simple to complex) that helps to inform whether anorectal manometry testing would be beneficial in evaluating incontinence [3].

Statistical analysis 

Baseline characteristics and self-reported practice were described using mean (±standard deviation), median (interquartile range), and frequencies/proportions as appropriate. Outcomes for continuous unpaired variables were analyzed with the nonparametric Mann-Whitney U test. Dichotomous outcomes were compared between groups using chi-square or Fisher’s exact tests with no adjustment for multiple comparisons. For the primary outcome, the relative comfort of performing a DRE was captured with the Likert scale and expressed as a proportion and 95% confidence interval. A secondary analysis of the primary outcome was performed using multivariate logistic regression to assess the contribution of confounding factors. The correlation between two quantitative variables was evaluated using Spearman’s rank correlation test. All analyses were performed using SPSS version 27 (IBM Corp., Armonk, NY, USA), and a two-tailed p-value <0.05 was considered statistically significant.

Permissions

This project was approved as a quality improvement project of negligible risk with authority to publish by the lead Human Research Ethics Sub-Committees on Safety, Quality Improvement and Governance (reference numbers: 28024, 28096). All participants provided informed consent for the publication of their de-identified data.

## Results

Demographics

The response rate was 27% (N = 452), with equal distribution of key demographics across all three health regions. Most respondents were postgraduate years one to four, with most being residents. Most respondents performed low volume amounts of DREs, with minimal amounts of DRE also completed during their education in medical school. High internal validity was demonstrated at 97.3% (Table 1).

**Table 1:** Demographic characteristics of respondent DiT. Values are the number of participants (%) unless otherwise indicated. DiT = doctor-in-training

Variable	Proportion	Number
N = 452	%	n
Health Service		
South Metropolitan	36.5	165
North Metropolitan	33.2	150
East Metropolitan	30.3	137
Gender		
Male	46.2	209
Female	49.3	223
Non-binary	4.4	20
Position		
Intern	28.8	130
Resident medical officer	44.7	202
Registrar	23	104
Fellow	3.5	16
Number of years post-graduate		
1 to 2 years	47.8	216
3 to 4 years	31.6	143
More than 5 years	20.6	93
Number of DRE performed in medical school		
0 to 5	70.8	320
6 to 10	15.9	72
11 or more	13.3	60
Number of DRE performed in the previous year		
0 to 20	65.5	296
More than 21	34.5	156
Passed internal validity question		
Yes	97.3	440
No	2.7	12

Training

Most respondents had prior DRE training, with most (71%) undergoing training during medical school. More than half of respondents felt that their DRE training needed to be improved, and most (64.4%) did not have a senior colleague independently verify their DRE findings during clinical practice (Table 2).

**Table 2:** DRE training and education characteristics of respondent DiTs. Values are the number of participants (%) unless otherwise indicated. DRE = digital rectal examination; DiT = doctor-in-training

Variable	Proportion	Number
N = 452	%	n
Received DRE training		
Yes	90.3	408
No formal training	9.7	44
Level of training when last received training for performing DRE		
Medical school	71	321
Internship	15.3	69
Residency	2	9
Registrar	2	9
No formal training	9.7	44
Opinion on the adequacy of DREtraining		
Adequate	45.6	206
Inadequate	54.4	246
Did a senior colleague independently verify DRE findings of DiTs?		
Yes	35.4	160
No	64.6	292

DRE practice

While most respondents would not perform a DRE as part of a routine medical examination, most would perform it as part of the assessment for functional issues such as anal incontinence or constipation. About 5% of respondents said they would never perform a DRE on patients (Table 3).

**Table 3:** Current DRE practice of respondent DiTs. Values are the number of participants (%) unless otherwise indicated. DRE = digital rectal examination; DiT = doctor-in-training

Variable	Proportion	Number
N = 452	%	n
Performs DRE as part of a routine medical examination		
Yes	36.7	166
No	63.3	286
Performs DRE for the presenting complaint of abdominal pain		
Yes	47.6	215
No	52.4	237
Performs DRE for the presenting complaint of anal incontinence		
Yes	68.1	308
No	31.9	144
Performs DRE for the presenting complaint of constipation		
Yes	60.4	273
No	39.6	179
Performs DRE sometimes for the presenting complaint of altered bowel habit		
Yes	46	208
No	54	244
Performs DRE always for the presenting complaint of altered bowel habit		
Yes	26.3	119
No	73.7	333
Will never perform a DRE on patients		
Yes	4.9	22
No	95.1	430

DRE components

While most of the respondents assessed the passive steps of inspection (98.2%), resting anal tone (76.8%), or assessment of the prostate (81.4%), a significant proportion rarely performed the functional DRE components that require active patient participation such as perineum straining. Almost all would inspect for stool/blood on the glove (98.6%) (Table 4).

**Table 4:** DRE practices of respondent DiTs. Values are the number of participants (%) unless otherwise indicated. DRE = digital rectal examination; DiT = doctor-in-training

Variable	Proportion	Number
N = 452	%	n
External inspection of perianal area		
Nearly always/Quite frequently	98.2	444
Sometimes	1.1	5
Rarely/Never	0.7	3
Observe perineum during straining		
Nearly always/Quite frequently	32.8	148
Sometimes	23.9	108
Rarely/Never	43.3	196
Test anocutaneous reflex		
Nearly always/Quite frequently	32.3	146
Sometimes	25	113
Rarely/Never	42.7	193
Assess anal resting tone		
Nearly always/Quite frequently	76.8	347
Sometimes	19.5	88
Rarely/Never	3.7	17
Palpate rectal wall for rectoceles and lesions		
Nearly always/Quite frequently	90.5	409
Sometimes	8.4	38
Rarely/Never	1.1	5
Assess prostate size and potential lesions		
Nearly always/Quite frequently	81.4	368
Sometimes	11.3	51
Rarely/Never	7.3	33
Palpate for prostate tenderness		
Nearly always/Quite frequently	76.5	346
Sometimes	16.6	75
Rarely/Never	6.9	31
Assess pelvic descend during straining		
Nearly always/Quite frequently	18.1	82
Sometimes	28.1	127
Rarely/Never	53.8	243
Assess anal sphincter and puborectalis lift		
Nearly always/Quite frequently	25.4	115
Sometimes	11.3	51
Rarely/Never	63.3	286
Palpate for levator ani tenderness		
Nearly always/Quite frequently	32.1	145
Sometimes	10.4	47
Rarely/Never	57.5	260
Inspect stool on glove		
Nearly always/Quite frequently	98.6	446
Sometimes	1.3	6
Rarely/Never	0	0

Only around half of the respondents were completely comfortable in performing a DRE (52.5%), and most respondents were confident in the diagnosis of mainly benign pathologies such as hemorrhoids (66.2%) or anal fissures (70.7%). There was a significant decline in confidence levels for diagnosing malignant or functional disorders (Table 5).

**Table 5:** DiT comfort and confidence in aspects of DRE. Values are the number of participants (%) unless otherwise indicated. DRE = digital rectal examination; DiT = doctor-in-training

Variable	Proportion	Number
N = 452	%	n
Comfortable in performing DRE		
Quite/Completely comfortable	52.5	237
Somewhat comfortable	29.2	132
Not comfortable	18.3	83
Diagnosing upper gastrointestinal bleed on DRE		
Very confident	60.8	275
Somewhat confident	34.3	155
Not confident	4.9	22
Assessing degree of prostatic enlargement on DRE		
Very confident	32.3	146
Somewhat confident	25	113
Not confident	42.7	193
Diagnosing rectal cancer on DRE		
Very confident	26.3	119
Somewhat confident	24.8	112
Not confident	48.9	221
Diagnosing prostate cancer on DRE		
Very confident	23.7	107
Somewhat confident	17.7	80
Not confident	58.6	265
Assessment of retroverted uterus on DRE		
Very confident	26.5	120
Somewhat confident	14.6	66
Not confident	58.9	266
Diagnosing pelvic floor dyssynergia on DRE		
Very confident	8.6	39
Somewhat confident	5.1	23
Not confident	86.3	390
Diagnosing levator-ani syndrome on DRE		
Very confident	10.4	47
Somewhat confident	4.4	20
Not confident	85.2	385
Diagnosing uncomplicated hemorrhoids on DRE		
Very confident	66.2	299
Somewhat confident	23.9	108
Not confident	9.9	45
Diagnosing anal fissures on DRE		
Very confident	70.7	320
Somewhat confident	25.7	116
Not confident	3.6	16
Diagnosing anal cancer on DRE		
Very confident	27.2	123
Somewhat confident	29.6	134
Not confident	43.2	195
Assessing anal sphincter weakness on DRE		
Very confident	45.8	207
Somewhat confident	31.2	141
Not confident	23	104

Univariate analysis

Univariate analysis demonstrated significant associations between comfort levels for performing DRE and seniority levels (for both position and number of years post-graduation with p = <0.001). Most DiTs in various specialties were comfortable performing the DRE except for those in General Medicine (25.4%). A significant positive association was also demonstrated in respondents who performed more DREs during medical school or the year preceding this study, with higher confidence levels in performing DRE as part of their clinical practice (Table 6).

**Table 6:** Univariate analysis results with the likelihood of DiTs being comfortable performing DRE as the dependent variable. Values are number of patients (%) unless otherwise indicated. Pearson chi-square analysis and Fisher’s exact test (for cell values <5), and bolded denotes significance at p < 0.05 SMHS, EMHS, NMHS = South, East, North Metropolitan Health Services; DiT = doctor-in-training; DRE = digital rectal examination

Variable (N = 452)		Comfort	Comfort	P-value
		(n)	(%)	
Region	SMHS	95	57.6%	0.252
	NMHS	74	49.3%	
	EMHS	68	52.4%	
Gender	Male	120	57.4%	0.001
	Female	114	51.1%	
	Non-binary	3	15.0%	
Position	Intern	53	40.8%	<0.001
	Resident	95	47.0%	
	Registrar	73	70.2%	
	Fellow	16	100.0%	
Post-graduate year (PGY)	PGY 1 and 2	94	43.5%	<0.001
	PGY 3 and 4	71	49.7%	
	PGY 5 and above	72	77.4%	
Last received DRE training	Medical school	194	60.4%	<0.001
	Internship	25	36.2%	
	Residency	9	100.0%	
	Registrar	2	22.2%	
	No training	7	15.9%	
Specialty interest group	General Surgery	65	64.4%	<0.001
	Urology	21	63.6%	
	Emergency Medicine	29	53.7%	
	Gastroenterology	12	50.0%	
	General Medicine	16	25.4%	
	General Practice	22	46.8%	
	Other surgical subspeciality	33	52.4%	
	Other medical subspecialty	39	58.2%	
Received DRE training	Yes	230	56.4%	<0.001
	No	7	15.9%	
Senior colleague verification	Yes	78	48.8%	0.246
	No	159	54.5%	
Number of DREs performed in medical school	0 to 5	151	47.2%	0.002
	6 to 10	46	63.9%	
	11 or more	40	66.7%	
Number of DREs performed in preceding year	0 to 20	111	37.5%	<0.001
	21 or more	126	80.8%	

Further, the univariate analysis also demonstrated significant positive associations between seniority levels and confidence in diagnosing malignant, benign, and functional conditions. All specialty subgroups were more confident in diagnosing benign conditions than malignant or functional ones. Of interest, the Emergency, General Practice, and General Medicine specialty interest groups had significantly less confidence in diagnosing conditions on DRE (Table 7).

**Table 7:** Univariate analysis results with the likelihood of DiTs being confident diagnosing conditions as the dependent variable. Values are the number of patients (%) unless otherwise indicated. Pearson chi-square analysis and Fisher’s exact test (for cell values <5), and bolded denotes significance at p < 0.05 SMHS, EMHS, NMHS = South, East, North Metropolitan Health Services; DiT = doctor-in-training; DRE = digital rectal examination

Variable (N = 452)		Malignant conditions			Benign conditions			Functional conditions		
		Comfort	Comfort	P-value	Comfort	Comfort	P-value	Comfort	Comfort	P-value
		(n)	(%)		(n)	(%)		(n)	(%)	
Region	SMHS	61	37.0%	0.177	132	80.0%	0.431	58	35.2%	0.002
	NMHS	42	28.0%		121	80.7%		82	54.7%	
	EMHS	40	29.2%		117	85.4%		67	48.9%	
Gender	Male	80	38.3%	0.002	178	85.2%	<0.001	85	40.7%	0.113
	Female	62	27.8%		183	82.1%		113	50.7%	
	Non-binary	1	5.0%		9	45.0%		9	45.8%	
Position	Intern	31	23.8%	<0.001	106	81.5%	0.011	52	40.0%	<0.001
	Resident	54	26.7%		155	76.7%		76	37.6%	
	Registrar	42	40.4%		93	89.4%		63	60.6%	
	Fellow	16	100.0%		16	100.0%		16	100.0%	
Post-graduate year (PGY)	PGY 1 and 2	45	20.8%	<0.001	178	82.4%	0.311	75	34.7%	<0.001
	PGY 3 and 4	38	26.6%		112	78.3%		64	44.8%	
	PGY >5	60	64.5%		80	86.0%		68	73.1%	
DRE training	Yes	143	35.0%	<0.001	326	79.9%	<0.001	198	48.5%	<0.001
	No	0	0.0%		44	100.0%		9	20.5%	
Last received DRE training	Medical school	109	34.0%	<0.001	273	85.0%	<0.001	156	48.6%	<0.001
	Internship	26	37.7%		35	50.7%		27	39.1%	
	Residency	6	66.7%		9	100.0%		6	66.7%	
	Registrar	2	22.2%		9	100.0%		9	100.0%	
	No training	0	0.0%		44	100.0%		9	20.5%	
Specialty interest group	General Surgery	53	52.5%	<0.001	82	81.2%	0.032	64	63.4%	<0.001
	Urology	24	72.7%		27	81.8%		24	72.7%	
	Emergency	15	27.8%		44	81.5%		18	33.3%	
	Gastroenterology	15	62.5%		24	100.0%		15	62.5%	
	General Medicine	4	6.3%		52	82.5%		20	31.7%	
	General Practice	6	12.8%		34	72.3%		18	38.3%	
	Other surgical subs	15	23.8%		46	73.0%		23	36.5%	
	Other medical subs	11	16.4%		61	91.0%		25	37.3%	
Senior verification	Yes	63	39.4%	0.009	119	74.4%	0.002	91	56.9%	<0.001
	No	80	27.4%		251	86.0%		116	39.7%	
Number of DREs performed in medical School	0 to 5	75	23.4%	<0.001	260	81.3%	0.787	117	36.6%	<0.001
	6 to 10	38	52.8%		61	84.7%		41	56.9%	
	11 or more	30	50.0%		49	81.7%		49	81.7%	
Number of DREs performed year	0 to 20	57	19.3%	<0.001	244	82.4%	0.663	110	37.2%	<0.001
	21 or more	86	55.1%		126	80.8%		97	62.2%	

Barriers and enablers

Respondents’ top three reasons for not performing DREs included being regarded as being too invasive (60%), lack of chaperone (59.3%), and lack of confidence in performing or interpreting findings (47.8%). Other less frequent reasons included a lesion that precludes a DRE, a patient’s age, anticipated patient refusal, the impression of little value/outdated practice, or the perception that DRE is just too much trouble. The key enablers of DRE performance were formal training and senior colleague/departmental support (Table 8).

**Table 8:** Reasons for respondent DiT not performing the DRE. Values are the number of participants (%) unless otherwise indicated. DiT = doctor-in-training; DRE = digital rectal examination

Rank	Variable	Proportion
N = 452		% of responses
1	Thought too invasive for routine examination	60.0%
2	Lack of chaperone	59.3%
3	Lack of confidence to perform or interpret findings	47.8%
4	Presence of a lesion that precludes a DRE	23.5%
5	Age of patient	14.6%
6	Anticipated patient refusal	13.5%
7	Limited value/Outdated practice	12.8%
8	Convenience concerns; can perform at colonoscopy	9.1%
9	Gender concern	7.7%
10	Too much trouble	6.0%
11	Religious or cultural convictions	4.6%

Multivariate analysis

**Figure 1:**
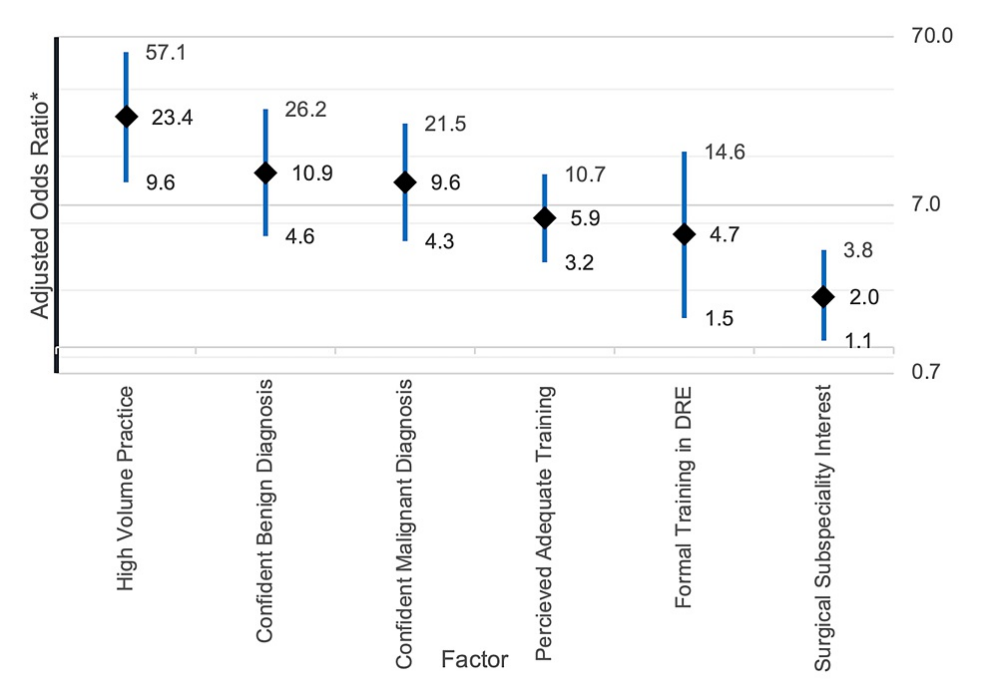
Multivariate logistic regression analysis with being comfortable in performing DRE as the dependent variable. DRE = digital rectal examination

Multivariate logistic regression controlled for statistically insignificant confounders and showed that clinicians who reported being comfortable in performing DRE were significantly and independently associated with being high-volume practitioners of DRE (OR = 23.4, 95% CI = 9.6-57.1, p < 0.001). Other significant factors which led to comfortable DRE clinical practice were having confidence in diagnosing benign pathology (OR = 10.9, 95% CI = 4.6-26.2, p < 0.001), confidence in diagnosing malignant pathology (OR = 9.6, 95% CI = 4.3-21.5, p < 0.001), perceived adequate training in DRE (OR = 5.9, 95% CI = 3.2-10.7, p < 0.001), prior formal training in DRE (OR = 4.7, 95% CI = 1.5-14.6, p = 0.007), and having surgical subspecialty interest (OR = 2.0, 95% CI = 1.1-3.8, p = 0.030) (Figure 1).

## Discussion

The DRE remains a useful physical examination component, providing valuable information for diagnosing multiple conditions across various specialties [1-4]. The lack of training and time, exacerbated by the lack of senior supervision and verification, could have a role in perpetuating the declining confidence in diagnosing anorectal conditions, further reducing its use in clinical medicine. The perception that DRE has low clinical yield may have reduced its usage in clinical practice. In addition, the ubiquity and overwhelming presence of sophisticated technological tools may be a causative factor worth considering. However, modern technology, such as radiological imaging and endoscopies, should not be the answer to clinical medicine.

The perceived invasiveness of DRE was reported as the most common reason for not performing DRE. While not extensively studied, previous studies have suggested that patients are not bothered by the procedure if the procedure and indications have been adequately described [9]. Patients’ perspectives on DRE could also be a focus for future studies in facilitating its ongoing use and uptake.

Subspecialty interest groups such as General Practice and Emergency Medicine should be encouraged to undergo training in this clinical tool as they are at the forefront of medicine. As the first point of contact for many patients, they are vital in ensuring that malignant pathologies are not missed or delayed. A missed opportunity could lead to devastating consequences for patients [5].

While respondents primarily performed the inspection and palpation aspects of DRE, other steps of the DRE that mainly involved evaluation of functional status were performed less. These included vital steps in assessing continence issues, such as evaluating pelvic descent during straining or anal sphincter and puborectalis lift. As suggested by previous studies, these steps may not have been adequately taught in medical school, translating to incomplete practice in the clinical field [8].

With the barriers identified, it is hoped that steps can be undertaken to improve the utility of the DRE in medical practice. The education of DRE should occur in medical school and be further cemented early in a junior doctor’s career to support their clinical skills. Implementing mannequins in medical school for various clinical skills is exceedingly common. High-fidelity mannequins with interchangeable pelvic and rectal pathologies could be used as introductory models for tactile skills in a workshop or simulation setting, with senior clinicians providing guidance and feedback. Training could be complemented with history and examination, integrating images with common anorectal pathologies to improve identification skills. As technology advances in medical education, virtual reality modules could also be helpful as this allows for real-time feedback from the module paired with clinician supervision.

Translating these skills into real-life practice is challenging due to the nature of the examination. A potential area that could be explored is the implementation of a skills session in an endoscopy or operating theater setting, thereby reducing the anxiety and perceived invasiveness from both the patients and DiTs. Although it needs to be highlighted with great caution that patients are fully informed and consent documented for this to occur.

There are several limitations of this study. First, the overall response rate was low at 27%. This is common for survey-based research involving health professionals [10-12]. This may result in a response bias in our study, where only individuals who felt strongly about the utility of the DRE or the level of DRE training they received would have responded to the questionnaire. In addition, the study only investigated the respondents’ self-reported clinical practice, with a lack of correlation between actual clinical skill and the reported confidence and comfort levels, which future studies might want to address.

Several strategies could be explored to mitigate the above limitations for future research. To improve response rates, reminders at various periods could be sent out to prompt responses or provide small incentives for completing the surveys. Another option is to liaise with the institutions’ education officers to promote completing such surveys at the start of continuous professional development sessions. Future research should also conduct a subgroup analysis of the different specialties in their attitudes and practices toward DRE. It will be informative to capture the responses from high-volume DRE practitioners from specialty fields compared to relatively low-volume ones. Outcomes learned from such targeted studies could serve as a foundation for developing specialty-specific training tools and practice guidelines to improve the utility of DRE.

## Conclusions

The position of DRE as a fundamental clinical tool has been debated in recent years with emerging technological advancements and reservations in its diagnostic yield from the self-sustaining cycle of lack of training and skills, leading to reduced confidence in its performance. DRE has limitations; however, when used in carefully selective presentations based on combined history and examination, it provides robust critical diagnostic information that can significantly impact patient management.

## Appendices

**Table 9:** Appendix 1 - Supplemental online materials - pipetted survey questionnaire

Survery Items
Page one
Project Title: Current Attitudes, Enablers and Barriers to Performing Digital Rectal Exam (DRE)
I hereby agree to be a participant in the above-named research.
Yes or No
Page two
Q1. Gender
0 = Male OR 1= Female OR 2 = Undeclared
Q2. What is your specialty of interest?
1 = General Surgery OR 2 = Urology OR 3 = Emergency Medicine OR 4 = Gastroenterology OR 5 = General Medicine OR 6 = Other Surgical Sub-Specialties OR 7 = Other Medical Sub-Specialties
Q3. What is your current rank?
1 = Intern OR 2 = Resident OR 3 = Registrar OR 4 = Fellow
Q4. How many years since you graduated from the school of Medicine?
Enter number_____ yy in years
Page three
Q5. How many DREs did you perform?
5.1 a. At the time of graduation from medical school?
Enter number_____ nn in Numbers.
5.2 b. In the last year?
Enter number_____ nn in Numbers.
Q6. How many DREs were refused by patients in the past year?
Enter number_____ nn in Numbers.
Rate of performances of the DRE in given five clinical scenarios
Q7. Given these clinical presentations, how likely are you to perform a DRE?
7.1. When chest discomfort is the presenting complaint (credibility question)
7.2. As part of a general medical examination
7.3. Where abdominal pain is the presenting complaint
7.4. Where faecal incontinence is the presenting complaint
7.5. Where constipation is the presenting complaint
Number as ______ (0–100) %
Steps in performing a DRE (rate of performance) five-point scale.
Q8. How often do you perform the following systematic steps of DRE?
8.1. External inspection of the perianal area
8.2. Watch perineum during straining
8.3. Test anal wink (anocutaneous reflex)
8.4. Assess resting anal tone
8.5. Palpate rectal wall for rectocele
8.6. Palpate prostate for size and sulcus preservation
8.7. Check pelvic descent during straining
8.8. Feel for anal sphincter and puborectalis lift
8.9. Palpate for levator-ani tenderness
8.10. Inspect stool on the finger
1. Nearly Always, 2. Quite Frequently, 3. Sometimes, 4. Rarely, 5. Never
Page four
Q9. How comfortable are you at performing a DRE? - (five-point scale)
1. Completely Comfortable, 2. Quite Comfortable, 3. Somewhat Comfortable, 4. A Little Bit Comfortable, 5. Not at All Comfortable
Confidence in DRE Clinical condition diagnosis
Q10. How confident are you at diagnosing the following conditions based on the DRE? (Clinical Conditions) five-point scale
0 = A GI bleed
1 = Degree of prostate enlargement
2 = Low rectal cancer
3 = Retroverted uterus
4 = Pelvic floor dys-synergia
5 = Levator ani syndrome
6 = Hemorrhoids
7 = Anal fissure
8 = Anal skin tag
9 = Anal cancer
10 = Anal sphincter weakness
11 = Anorectal stricture
1. Completely Confident, 2. Quite Confident, 3. Somewhat Confident, 4. A Little Bit Confident, 5. Not at All Confident
Q11. What are your three most frequent reasons for not performing/initiating a DRE?
0 = Concern for patient’s modesty OR 1 = Age of patient OR 2 = Limited value, outdated, not useful OR 3 = Concern for my own modesty OR 4 = Anticipated patient refusal OR 5 = Religious/Cultural Convictions OR 6 = Not indicated: patient has no abdominal pain OR 7 = Need confidence/competence to do it OR 8 = Gender concern OR 9 = Too much trouble OR 10 = Too invasive for routine exam OR 11 = Convenience: can do at colonoscopy OR 12 = No specific reason
Page five - Training
Q12. When was the DRE technique last formal teaching?
0 = Internship OR 1 = Residency years OR 2 = Registrar years OR 3 = Fellowship OR 4 = Never Taught
Q13. Was the training you received on how to perform a DRE adequate?
0 = No OR 1 = Yes
Q14. If still doctor-in-training, how often does a senior colleague confirm your findings by performing their own independent DRE? (five-point scale)
1. Nearly Always, 2. Quite Frequently, 3. Sometimes, 4. Rarely, 5. Never
Q15. What improvements would you like to see implemented to the training curricula?
[Free Text Box]
Page six
Q16. I would you like to receive an executive summary of the research findings
0 = Yes or 1 = No
Q17. If you answered yes to the above question, please provide your preferred email address for correspondence:
[Free Text Box]
The contact details provided will be extracted separately from the study data to maintain anonymity
Please note that this research group takes your confidentiality very seriously
At no time will the raw data collected in this survey be released to any third party
Thank you for taking your time to complete this survey, it is greatly appreciated!
End of the survey
https://www.surveymonkey.com/r/DREWA
